# Factors influencing compassion satisfaction and compassion fatigue among nurses: a study in a tertiary hospital

**DOI:** 10.1186/s12912-025-02736-3

**Published:** 2025-01-27

**Authors:** Sarah Sharmala Nadarajan, Ping Lei Chui, Wan Ling Lee, Noor Hanita Zaini

**Affiliations:** Department of Nursing Science, Faculty of Medicine, University of Malaya, Kuala Lumpur, 50603 Malaysia

**Keywords:** Burnout, Compassion fatigue, Risk factors, Workplace environment, Nursing

## Abstract

**Background:**

Nursing is a caring profession for which compassion is a core value. Increasing stress and declining job satisfaction are among the major challenges in nursing. Demographic and work-related factors may influence nurses’ compassion satisfaction and compassion fatigue (i.e., burnout and secondary traumatic stress) levels. In this study, the level of compassion fatigue and compassion satisfaction and their associated factors were examined among nurses in a tertiary hospital.

**Methods:**

A cross-sectional study was conducted at a tertiary hospital in Malaysia. The data were collected over a period of 6 months via online distribution of the Personal Information Form, Copenhagen Psychosocial Questionnaire (COPSOQ) version III and Professional Quality of Life (ProQOL) version V questionnaires. The Cronbach’s alpha internal consistency of the questionnaire scales was mostly acceptable and above 0.75. Descriptive statistics were used to summarize the sociodemographic and rank domains of work environment-related factors for nurses and their levels of compassion satisfaction and compassion fatigue. Relationships between sociodemographic factors and the levels of compassion fatigue, compassion satisfaction, and burnout were assessed by bivariate analyses. A *p* value < 0.05 was considered to indicate statistical significance.

**Results:**

A total of 323 registered nurses participated in this study. A majority of the participants were female (91%, *n* = 294), and the mean age of the participants was 33.01 ± 8.50 years. The majority of the participants had moderate levels of compassion satisfaction (71%, *n* = 229); 46% (*n* = 148) had moderate levels of burnout, and 45% (*n* = 147) had moderate levels of secondary traumatic stress. Hierarchical multiple regression analysis revealed that the health and well-being and demands at work domain were significantly associated with compassion satisfaction, burnout and secondary traumatic stress levels among nurses.

**Conclusions:**

In this study, the majority of the nurses reported decreased compassion satisfaction and increased burnout. These findings provide valuable insights, as there may be detrimental effects on the healthcare industry and retention of nurses if no action is taken to combat compassion fatigue. Recommendations to motivate nurses and reduce demands at work should be explored by healthcare organizations to increase nurses’ performance and job satisfaction.

## Introduction

### Background

Nurses play a vital role in the healthcare system. Fundamental indicators related to their contentment include job satisfaction and high-quality work life. Nurses are professionals, and providing care in a compassionate manner is one of the attributes of a nurse. The professional quality of life (ProQOL) of a nurse comprises a positive emotional state described as compassion satisfaction and a negative emotional state described as compassion fatigue [1]. Compassion satisfaction is desirable in the interaction between a patient and a nurse since it gives nurses a sense of contentment from caring for others, such as understanding the patient’s perspective, providing emotional support and advocating for their needs. Compassion fatigue is described as the depletion of energy and self-blaming in the process of providing care when one is unable to protect or heal the patient from suffering [2]. Prior studies have focused on burnout and secondary traumatic stress as components of compassion fatigue [3]. Nurses with higher levels of compassion satisfaction have a lower risk of experiencing compassion fatigue [4].

The degrees of compassion satisfaction and compassion fatigue are significantly related to the personal characteristics of nurses, including age, marital status, level of education, religious affiliation, and nursing position [5]. Work environment factors such as workload and job demand, work‒life balance, social support at the workplace, shift length and reward influence nurses’ compassion satisfaction, burnout and secondary traumatic stress level [6–9]. Cultural and religious variations across regions also influence burnout and secondary traumatic stress levels among nurses [10].

The high attrition rate among nurses is attributed to compassion fatigue [11, 12]. Similarly, Malaysia was predicted to experience a nursing shortage by 2020 [13]. According to the findings of studies conducted in Malaysia, nurses reported low to moderate levels of job satisfaction related to support from supervisors, salary, and nursing management policies [14]. Before these issues can be addressed, it is imperative that the current level of compassion satisfaction and compassion fatigue and their associated factors are identified among nurses in Malaysia. Therefore, the objectives of this study were (i) to determine compassion satisfaction and compassion fatigue levels among nurses and (ii) to identify factors (i.e., personal and work environment-related factors) that are significantly associated with compassion satisfaction and compassion fatigue among nurses. This study was guided by Stamm’s compassion satisfaction and compassion fatigue model [15], which suggests that personal and work environment-related factors predict nurses’ compassion satisfaction and compassion fatigue levels. Personal factors include nurses’ personal information, such as age and sex, whereas work environment-related factors are other factors in addition to personal information, such as the workload of nurses and the working environment. Hence, this framework was used in this study to assist in identifying factors that influence nurses’ compassion satisfaction and compassion fatigue.

## Methods

### Design and setting

The aim of this descriptive, cross-sectional study was to determine the level of compassion fatigue and compassion satisfaction and their associated factors among nurses in a tertiary hospital. The study was conducted among registered nurses in a tertiary hospital in Malaysia between November 2020 and May 2021. The STROBE checklist was utilized as a framework for transparent and comprehensive reporting of this study [16].

### Sample and population

The teaching hospital has 1623 beds, an average patient stay of 4.92 days, and 28 different wards and units with various specialties. It has catered to 1,171,648 service recipients from the outpatient department and 55,756 service recipients from the inpatient units. The hospital employs more than 2300 nurses, and the nurse-to-patient ratio in critical care units is 1:2–4, whereas in medical surgical wards, the nurse-to-patient ratio is 1:8–10. The research population consisted of registered nurses who worked in any of the 3 areas categorized as the critical care unit, the medical-surgical unit, and other nursing services, such as ambulatory units. These three areas of nursing services are responsible for providing the clinical, managerial and educational resources necessary to support nurses in accordance with established professional standards. Registered nurses are nurses who have obtained a diploma in nursing, a 3-year nondegree nursing program from a nursing college that will enable a student to become a registered nurse upon completion of the course and who has successfully passed the Malaysia Nursing Board examination. Nurses begin shifts at 7am and end at 2 pm for morning shifts. They work from 2 to 9 pm for the afternoon shift and from 9 pm to 7am the following day for the night shift. Nurses, mainly from ambulatory units, work office hours from 8 am to 5 pm or from 8:30 am to 5:30 pm. The sample was selected on the basis of the following inclusion criteria: registered nurses working in the hospital who participate directly in patient care. Moreover, nursing students and non-nursing personnel, such as healthcare attendants and other healthcare providers, were excluded from this study. The sample size was calculated using the G*Power software (version 3.1.9.2), and the calculation was based on an alpha of 0.05 and a power of 80%, whereas effect sizes were estimated from previous similar studies by Wang et al. (2020); the recommended minimum sample size was 319 participants [17].

### Data collection procedure

The researcher distributed the questionnaire to the sample population until the sample size was reached, considering the inclusion and exclusion data. After permission was obtained from the hospital’s director of nursing, a brief explanation of the study's purpose was given. The self-administered questionnaire was distributed through an online platform (Google form) where each participant was given a URL link via e-mail. The questionnaires were distributed to the nurses at the beginning of their duty shift. The respondents provided their consent to participate in the study by clicking the ‘Agree’ button in the Google form. The participants were provided approximately 20 to 30 min to complete the questionnaire. Some of the items are set as open-ended questions to avoid bot-generated responses. The collected data were reviewed for missing information to ensure that they were complete. The participants were assured that their confidentiality and anonymity would be ensured throughout the study.

### Instruments

The study used three data collection tools: the ‘Personal Information Form’ was used to obtain nurses’ demographic characteristics, the ‘Copenhagen Psychosocial Questionnaire (COPSOQ) Version III’ was used to gather information on the nurses’ work environment, [18] and the ProQOL was used to measure the nurses’ level of compassion satisfaction, burnout, and secondary traumatic stress [19]. The questionnaire was administered in English and Malay. The questionnaire was translated into Malay by two independent translators, and those versions were later merged into a single version by the research team. The questionnaire was translated back to English by another independent translator whose native language was Malay. The translator had no knowledge of the original questionnaire. All the versions were subsequently reviewed and compared to the original version by the research team, and the Cronbach’s alpha internal consistency reliability of the questionnaire was assessed.

#### Demographic characteristics

The demographic characteristics consisted of 15 sociodemographic items, such as age, sex, marital status, highest attained qualification, years of working experience as a registered nurse, reason to join nursing, participant’s work schedule, requirement of working overtime or double shifts in a month, adequate staffing, requirement to work night shifts, current designation, any medical leave taken for the past six months, any current health problems that they may experience and the possibility of working as a nurse for the next five years. The demographic variables were adapted mainly from previous studies on compassion fatigue and compassion satisfaction [20–22]. A few other items, such as the reason for becoming a nurse, were also included as demographic variables to determine whether personal choice in pursuing nursing as a career influences nurses’ compassion fatigue and compassion satisfaction level.

#### COPSOQ version III

The COPSOQ version III was adapted from Useche et al. (2019) to assess nurses’ work environment, including psychosocial factors [18]. The self-answered questionnaire contains 60 items categorized into six domains (demands at work, 11 items; work organization and job content, 13 items; interpersonal relations and leadership, 18 items; work‒individual interface, 11 items; social capital, 6 items; and health and well-being, 1 item). The results of the six domains of work environment-related factors are presented as the means and standard deviations. Content validity was assessed by an expert panel consisting of lecturers from the Department of Nursing Science and nursing managers from the Nursing Department to ensure the quality and relevance of the items. The instrument has a Cronbach’s alpha of 0.72 and good internal reliability [23].

#### ProQOL

The ProQOL version V was developed by Stamm (2009) and was used to measure work-related QOL among nurses. It is a 30-item questionnaire with a 5-point Likert scale (ranging from 1 = never to 5 = very often) [24]. The questionnaire consists of three sets of 10 items that reflect specific measures on the ProQOL subscales: compassion fatigue, burnout, and secondary traumatic stress. The total score of each subscale was interpreted as high (≥ 42), moderate (23–41), or low (≤ 22). A higher score indicates a high level of the components being measured. Past studies have demonstrated that the ProQOL scale has good internal consistency reliability, with Cronbach’s alpha values of 0.80 for burnout, 0.72 for secondary traumatic stress, and 0.87 for compassion satisfaction [25]. In this study, the Cronbach’s alpha internal consistency values of the questionnaire scales were mostly acceptable and above 0.75. The internal consistency reliability of Cronbach’s alpha for the COPSOQ among registered nurses was 0.80 and above, whereas the internal consistency reliability for the ProQOL scores was 0.76 and above. The Cronbach’s alpha for the health and well-being domain of the COPSOQ could not be examined because there was only one item in the domain.

### Ethical consideration

Research was performed in accordance with the World Medical Association’s Declaration of Helsinki and was approved by the Medical Research Ethics Committee (MRCEID NO. 202075–8864) where the study was conducted. Confidentiality and anonymity were assured to the participants, and they were informed of the right to withdraw from the study at any time without any negative consequences for their employment.

### Data analysis

All the statistical analyses were carried out with SPSS software version 23.0 (IBM Corp., Armonk, NY, USA). Categorical variables (i.e., demographic characteristics, compassion satisfaction and compassion fatigue) are presented as frequencies and percentages. Continuous variables (i.e., work environment-related factors of nurses) are presented as the means and standard deviations, and 95% confidence intervals were calculated. Descriptive statistics were used to summarize sociodemographic characteristics and compassion satisfaction and compassion fatigue levels, and the relationships between sociodemographic and work-related characteristics and the levels of compassion fatigue, compassion satisfaction, and burnout were assessed by independent-samples t tests, one-way ANOVA and hierarchical linear regression. A *p* value < 0.05 was considered to indicate statistical significance.

## Results

### Sociodemographic characteristics of the registered nurses

The participants’ characteristics are shown in Table 1. Three hundred and twenty-three participants were involved in this study, and their mean age was 33.01 ± 8.50 years. The majority of the participants in this study were women (*n* = 294, 91%), were married (*n* = 216, 67%), and had a diploma in nursing (*n* = 181, 56%). The detailed characteristics of the participants are shown in Table 1.

**Table 1:** Socio-demographic characteristics of Registered Nurse (*n* = 323)

Variables	*n (%)*	*Mean (SD)*
**Age (in years)**		33.01 (8.50)
22 to 30	143 (44)	
31 to 40	122 (38)	
41 and above	58 (18)	
**Gender**		
Male	29 (9)	
Female	294 (91)	
**Marital Status**		
No spouse (single/widow/divorcee)	107 (33)	
Living with spouse (married)	216 (67)	
**Reason to Join Nursing**		
Self-interest	202 (63)	
Family Motivation	104 (32)	
Others	17 (5)	
**Highest Attained Qualification**		
Diploma	181 (56)	
Post Basic and above	142 (44)	
**Working Experience as a Nurse**		10.83 (7.71)
≤ 10 years	189 (59)	
> 10 years	134 (41)	
**Current Working Department**		
Critical Care Unit^a^	73 (23)	
Medical Surgical Unit^b^	176 (54)	
Others^c^	74 (23)	
**Working Schedule**		
Shift Work	232 (72)	
Office Hours	91 (28)	
**Requirement to Do Night Shifts**		
Yes	256 (79)	
No	67 (21)	
**Working with Adequate Staffing**		
Yes	145 (45)	
No	178 (55)	
**Required to Work Over Time/Double Shift**		
Yes	120 (37)	
No	203 (63)	
**Experiencing Medical Conditions/Illnesses**		
Yes	196 (61)	
No	127 (39)	
**Medical Leave Taken for the Past 6 Months**		
Yes	175 (54)	
No	148 (46)	
**Likely to Work as a Nurse for the Next 5 Years**		
Yes	236 (73)	
Ambiguous (No/Maybe)	87 (27)	

^a^Inclusive of Accident & Emergency Unit & Intensive Care Units

^b^Inclusive of adult and paediatric Medical Surgical Units

^c^Inclusive of Operating Theatre & Ambulatory Units

### Ranking of the work environment-related factors of nurses by domain

Six domains involving work environment-related factors were assessed in this study via the COPSOQ version III (Table 2). The work organization and job content domain yielded the highest mean score of 65.50 (SD = 9.23), followed by the health and well-being domain (M = 62.15, SD = 20.98), the interpersonal relations and leadership domain (M = 61.86, SD = 10.46), the work‒individual interface domain (M = 61.73, SD = 14.77), the social capital domain (M = 61.12, SD = 16.31) and the demands at work domain (M = 51.14, SD = 15.91).

**Table 2:** Rank of domains the work environment-related factors of nurses (*N* = 323)

Work Environment Variables	*Mean (SD)*
Work Organization and Job Contents Domain	65.50 ± 9.23
Health and Well-being Domain	62.15 ± 20.98
Interpersonal Relations and Leadership Domain	61.86 ± 10.46
Work-Individual Interface Domain	61.73 ± 14.77
Social Capital Domain	61.12 ± 16.31
Demands at Work Domain	51.14 ± 15.91

### Levels of compassion satisfaction and fatigue

**Fig. 1:**
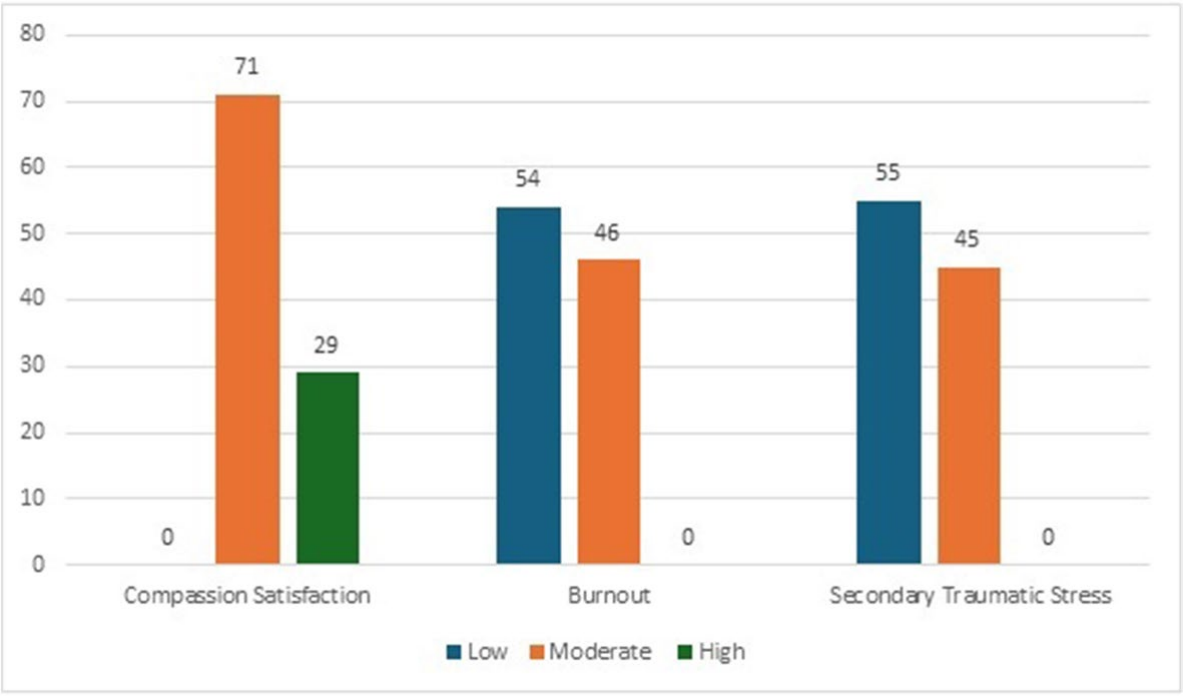
Nurses’ level of compassion satisfaction and compassion fatigue (burnout and secondary traumatic stress) (*N* = 323). Low = score = 22; Moderate = score 23–41; High = score 42

Nurses’ levels of compassion satisfaction and fatigue were measured using the ProQOL scale Version V. The mean scores for compassion satisfaction, burnout, and secondary traumatic stress were 38.48 (SD = 5.50), 22.28 (SD = 5.20) and 22.19 (SD = 5.17), respectively. Although 71% of the nurses (*n* = 229) had moderate levels of compassion satisfaction, 46% (*n* = 148) had moderate levels of burnout, and 45% (*n* = 147) had moderate levels of secondary traumatic stress, as shown in Fig. 1. None of the nurses reported low levels of compassion satisfaction or high levels of burnout and secondary traumatic stress.

### Correlation between compassion satisfaction and compassion fatigue (burnout and secondary traumatic stress)

Pearson correlation coefficients were calculated to evaluate the relationships among compassion satisfaction, burnout, and secondary traumatic stress. Compassion satisfaction was negatively but strongly correlated with burnout (*r* = −0.772, *p* < 0.001) and negatively but weakly correlated with secondary traumatic stress (*r* = −0.387, *p* < 0.001). In contrast, burnout was positively and moderately correlated with secondary traumatic stress (*r* = 0.620, *p* < 0.001).

### Factors predicting compassion satisfaction, burnout and secondary traumatic stress

Two-step hierarchical regression analysis was used to determine the factors contributing to compassion satisfaction and compassion fatigue (burnout and secondary traumatic stress). Prior to conducting the hierarchical regression analysis for compassion satisfaction and compassion fatigue (burnout and secondary traumatic stress), a preliminary analysis was performed to evaluate the assumption. The sociodemographic characteristics of the nurses were entered into the first block analysis, after which all six domains from the COPSOQ version III were added to the existing sociodemographic characteristics of the nurses in the second block analysis.

Thirteen sociodemographic variables at an alpha level of 0.25 or less were identified in the bivariate analysis as potential covariates to predict compassion satisfaction using hierarchical multiple regression analysis, as shown in Table 3. In the first block analysis, the model accounted for 29.5% of the variation in nurses’ compassion satisfaction levels (*F* (15, 307) = 8.560, *p* < 0.001, *R*^*2*^ = 0.295). However, in the second block analysis, the model accounted for an additional 16.2% of the variation in the nurses’ compassion satisfaction levels (Δ*F* (6, 301) = 15.024, *p* < 0.001, *R*^*2*^ = 0.457). Nurses’ marital status, current working department, interpersonal relations and leadership domain and demands at work domain were statistically significant predictors of nurses’ compassion satisfaction levels (*p* < 0.05), whereas the work organization and job content domain as well as the health and well-being domain were highly statistically significant predictors of nurses’ compassion satisfaction level (*p* < 0.001).

**Table 3:** Hierarchical regression analysis for variables predicting compassion satisfaction

**Predictor**	**Model 1**					**Model 2**				
	**b**	**β**	**95% CI**		**t**	**b**	**β**	**95% CI**		**t**
			***LL***	***UL***				***LL***	***UL***	
**(Constant)**	38.301		34.480	42.121	19.725**	23.145		17.241	29.048	7.715**
Age	0.071	0.110	−0.060	0.202	1.069	0.068	0.106	−0.052	0.188	1.122
Gender; Male **(Female)**	−2.416	−0.126	−4.345	−0.488	−2.466*	−1.404	−0.073	−3.151	0.343	−1.582
Marital Status; Single **(Married)**	−1.980	−0.170	−3.214	−0.745	−3.156*	−1.493	−0.128	−2.595	−0.391	−2.667*
Reason to Join Nursing; Family Motivation **(Self-interest)**	−0.659	−0.056	−1.822	0.504	−1.115	−0.522	−0.044	−1.557	0.513	−0.993
Reason to Join; Others **(Self-interest)**	−2.406	−0.098	−4.831	0.019	−1.952	−0.701	−0.029	−2.924	1.522	−0.621
Highest Attained Qualification; Post Basic and above **(Diploma)**	−0.424	−0.038	−1.979	1.130	−0.537	−0.240	−0.022	−1.630	1.151	−0.339
Designation Grade; U32 **(U29)**	2.003	0.167	−0.116	4.123	1.860	1.680	0.140	−0.216	3.576	1.744
Years of Working Experience; >10yrs **(0–10 years)**	−1.786	−0.160	−3.785	0.214	−1.757	−1.488	−0.134	−3.296	0.320	−1.620
Likely to Continue Working as a Nurse; No **(Yes)**	−2.397	−0.194	−3.633	−1.161	−3.815**	−1.040	−0.084	−2.180	0.100	−1.795
Current Working Department; Critical Care Unit **(Medical Surgical Unit)**	−1.898	−0.145	−3.259	−0.538	−2.746*	−1.601	−0.122	−2.836	−0.366	−2.552*
Current Working Department; Others **(Medical Surgical Unit)**	−2.195	−0.168	−3.580	−0.809	−3.117*	−2.076	−0.159	−3.310	−0.843	−3.312*
Working Schedule; Office Hours **(Shift Work)**	0.343	0.028	−1.658	2.344	0.337	0.586	0.048	−1.206	2.377	0.643
Required to do Night Shifts; No **(Yes)**	−0.243	−0.018	−2.200	1.714	−0.244	0.307	0.023	−1.445	2.058	0.345
Adequate Staffing; Yes **(No)**	2.090	0.189	0.948	3.232	3.601**	0.827	0.075	−0.280	1.933	1.470
Working Overtime Double Shift; Yes **(No)**	−0.093	−0.008	−1.215	1.030	−0.163	0.697	0.061	−0.314	1.709	1.356
Work Organization and Job Contents Domain						0.126	0.211	0.066	0.186	4.144**
Health and Well-being Domain						0.057	0.217	0.032	0.081	4.586**
Interpersonal Relations and Leadership Domain						0.067	0.128	0.007	0.127	2.194*
Work Individual Interface Domain						0.027	0.072	−0.008	0.062	1.504
Social Capital Domain						−0.002	−0.005	−0.038	0.035	−0.084
Demands at Work						−0.061	−0.175	−0.097	−0.024	−3.253*
	**Model Summary:** Δ*F *(15, 307)* = *8.560, *p *<0.001					Δ*F* (6, 301)* =* 15.024, *p *<0.001				
	*R *= 0.543*, R*^*2*^ = 0.295					* R *= 0.676, *R*^*2*^ = 0.457				
						Δ*R*^*2*^ = 0.162				

Reference category are in bold and in bracket;*p <0.05; **p <0.001

Fourteen sociodemographic variables at an alpha level of 0.25 or less were identified as potential covariates for predicting burnout using hierarchical multiple regression analysis, as shown in Table 4. The first block analysis revealed that the model accounted for 33.1% of the variation in nurses’ burnout level (*F* (16, 306) = 9.459, *p* < 0.001, *R*^*2*^ = 0.331). In the second block analysis, the model accounted for 52.8% of the variation in nurses’ burnout level (Δ*F* (6, 300) = 20.929, *p* < 0.001, *R*^*2*^ = 0.528). Nurses’ marital status, current working department and social capital domain were statistically significant predictors of nurses’ burnout level (*p* < 0.05), whereas the work organization and job content domain, the health and well-being domain and the demands at work domain were statistically incredibly significant predictors of nurses’ burnout level (*p* < 0.001).

**Table 4:** Hierarchical regression analysis for variables predicting burnout

**Predictor**	**Model 1**					**Model 2**				
	**b**	**β**	**95% CI**		**t**	**b**	**β**	**95% CI**		**t **
			***LL***	***UL***				***LL***	***UL***	
**(Constant)**	25.098		21.443	28.753	13.511**	28.924		23.677	34.171	10.847**
Age	−0.114	−0.186	−0.235	0.008	−1.839	−0.043	−0.071	−0.151	0.064	−0.799
Gender; Male **(Female)**	2.605	0.143	0.823	4.387	2.877*	1.214	0.067	−0.333	2.760	1.544
Marital Status; Single **(Married)**	2.102	0.190	0.953	3.250	3.601**	1.496	0.136	0.510	2.482	2.985*
Reason to Join Nursing; Family Motivation **(Self-interest)**	0.451	0.041	−0.624	1.525	0.825	0.303	0.027	−0.612	1.218	0.652
Reason to Join; Others **(Self-interest)**	1.828	0.079	−0.423	4.080	1.598	−0.027	−0.001	−1.994	1.941	−0.027
Highest Attained Qualification; Post Basic and above **(Diploma)**	0.795	0.076	−0.641	2.231	1.090	0.280	0.027	−0.950	1.509	0.448
Designation Grade; U32 **(U29)**	−1.023	−0.090	−2.981	0.934	−1.029	−0.431	−0.038	−2.107	1.245	−0.506
Years of Working Experience; >10yrs **(0–10 years)**	0.883	0.084	−0.964	2.729	0.941	0.258	0.024	−1.342	1.857	0.317
Experiencing Medical Condition / Illness No **(Yes)**	−1.928	−0.181	−2.993	−0.862	−3.559**	−0.352	−0.033	−1.314	0.610	−0.720
Likely to Continue Working as a Nurse; No **(Yes)**	1.585	0.135	0.434	2.736	2.710*	0.598	0.051	−0.412	1.608	1.165
Current Working Department; Critical Care Unit **(Medical Surgical Unit)**	1.399	0.113	0.136	2.662	2.179*	1.067	0.086	−0.029	2.163	1.915*
Current Working Department; Others **(Medical Surgical Unit)**	1.761	0.142	0.481	3.042	2.706*	1.602	0.130	0.511	2.692	2.890*
Working Schedule; Office Hours **(Shift Work)**	0.256	0.022	−1.594	2.105	0.272	0.293	0.025	−1.292	1.879	0.364
Required to do Night Shifts; No **(Yes)**	−0.244	−0.019	−2.056	1.567	−0.265	−0.450	−0.035	−1.999	1.100	−0.571
Adequate Staffing; Yes **(No)**	−2.549	−0.244	−3.606	−1.493	−4.748**	−0.715	−0.068	−1.694	0.263	−1.439
Working Overtime Double Shift; Yes **(No)**	0.247	0.023	−0.793	1.287	0.467	−0.405	−0.038	−1.300	0.491	−0.890
Work Organization and Job Contents Domain						−0.091	−0.162	−0.144	−0.038	−3.394**
Health and Well-being Domain						−0.046	−0.187	−0.068	−0.024	−4.139**
Interpersonal Relations and Leadership Domain						−0.033	−0.067	−0.087	0.020	−1.236
Work Individual Interface Domain						0.021	0.061	−0.010	0.053	1.338
Social Capital Domain						−0.036	−0.113	−0.069	−0.004	−2.183*
Demands at Work						0.111	0.338	0.078	0.143	6.711**
	**Model Summary:** Δ*F *(16, 306) *= *9.459, *p *<0.001					Δ*F *(6, 300) *=* 20.929, *p *<0.001				
	*R *= 0.575, *R*^*2*^ = 0.331					*R *= 0.727, *R*^*2*^ = 0.528				
						Δ*R*^*2*^ = 0.197				

Reference category are in bold and in bracket;*p <0.05; **p <0.001

Thirteen sociodemographic variables at an alpha level of 0.25 or less were identified as potential covariates for the prediction of secondary traumatic stress using hierarchical multiple regression analysis, as shown in Table 5. The first block analysis revealed that the model accounted for 13.9% of the variation in nurses’ secondary traumatic stress level (*F* (15, 307) = 3.312, *p* < 0.001, *R*^*2*^ = 0.139). In the second block analysis, the model accounted for 10.7% of the variation in nurses’ secondary traumatic stress level (Δ*F* (6, 301) = 7.137, *p* < 0.001, *R*^*2*^ = 0.246). The health and well-being domain and the work individual interface domain were statistically significant predictors of nurses’ secondary traumatic stress level (*p* < 0.05), whereas the demands at work domain were very statistically significant predictors of nurses’ secondary traumatic stress level (*p* < 0.001).

**Table 5:** Hierarchical regression analysis for variables predicting secondary traumatic stress

** Predictor**	**Model 1**					**Model 2**				
	**b**	**β**	**95% CI**		**t**	**b**	**β**	**95% CI**		**t**
			***LL***	***UL***				***LL***	***UL***	
**(Constant)**	23.729		19.740	27.717	11.706**	16.833		10.411	23.254	5.158**
Age	−0.015	−0.025	−0.147	0.116	−0.230	0.026	0.043	−0.104	0.156	0.398
Gender; Male **(Female)**	2.020	0.112	0.045	3.995	2.013*	1.165	0.064	−0.758	3.089	1.192
Marital Status; Single **(Married)**	0.857	0.078	−0.446	2.160	1.295	0.573	0.052	−0.675	1.821	0.904
Reason to Join Nursing; Family Motivation **(Self-interest)**	0.509	0.046	−0.701	1.719	0.828	0.396	0.036	−0.752	1.545	0.679
Reason to Join; Others **(Self-interest)**	1.703	0.074	−0.820	4.226	1.328	1.252	0.054	−1.212	3.716	1.000
Designation Grade; U32 **(U29)**	−0.370	−0.033	−2.564	1.823	−0.332	0.115	0.010	−1.977	2.207	0.108
Years of Working Experience; >10yrs **(0–10 years)**	0.097	0.009	−1.827	2.020	0.099	−0.562	−0.054	−2.421	1.296	−0.595
Experiencing Medical Condition / Illness; No **(Yes)**	−1.127	−0.107	−2.355	0.102	−1.805	−0.136	−0.013	−1.364	1.093	−0.217
Any Medical Leave Taken for The Past 6 Months; No **(Yes)**	−0.641	−0.062	−1.774	0.492	−1.113	−0.447	−0.043	−1.530	0.635	−0.813
Likely to Continue Working as a Nurse; No **(Yes)**	0.336	0.029	−0.959	1.632	0.511	−0.118	−0.010	−1.384	1.147	−0.184
Current Working Department; Critical Care Unit **(Medical Surgical Unit)**	0.501	0.041	−0.913	1.915	0.698	0.125	0.010	−1.244	1.494	0.180
Current Working Department; Others **(Medical Surgical Unit)**	0.239	0.019	−1.177	1.656	0.332	0.024	0.002	−1.321	1.369	0.035
Working Schedule; Office Hours **(Shift Work)**	−0.364	−0.032	−2.435	1.707	−0.346	−0.067	−0.006	−2.046	1.913	−0.066
Required to do Night Shifts; No **(Yes)**	−0.251	−0.020	−2.271	1.768	−0.245	−0.249	−0.020	−2.177	1.679	−0.254
Adequate Staffing; Yes **(No)**	−2.336	−0.225	−3.518	−1.154	−3.890**	−1.172	−0.113	−2.394	0.050	−1.887
Work Organization and Job Contents Domain						−0.038	−0.068	−0.105	0.028	−1.132
Health and Well-being Domain						−0.031	−0.126	−0.059	−0.003	−2.201*
Interpersonal Relations and Leadership Domain						0.015	0.031	−0.051	0.082	0.457
Work Individual Interface Domain						0.066	0.187	0.026	0.105	3.280*
Social Capital Domain						0.006	0.019	−0.035	0.047	0.293
Demands at Work						0.080	0.247	0.040	0.120	3.932**
	**Model Summary:** Δ*F *(15, 307) *= *3.312,* p *<0.001					Δ*F *(6, 301) *=* 7.137, *p *<0.001				
	*R *= 0.373, *R*^*2*^ =0.139					*R *= 0.496, *R*^*2*^ = 0.246				
						Δ*R*^*2*^ = 0.107				

Reference category are in bold and in bracket; *p <0.05; **p <0.001

## Discussion

This study was aimed at determining the levels of compassion fatigue and compassion satisfaction and their associated factors among registered nurses at a tertiary hospital. Nurses with many years of working experience have a sense of control over their work, as they are likely not novices to the expected tasks assigned at the workplace. Most nurses experienced moderate levels of satisfaction in their work and with the help that they provided to others. They did not harbour alarming levels of feeling overwhelmed by their work or experienced any fear in association with their work. The outcome of this study is similar to that of a study conducted by Kim et al. (2015) in Korea [5].

The negative correlation between compassion fatigue and compassion satisfaction and the positive correlation between burnout and secondary traumatic stress are evidence that compassion fatigue levels decrease with increasing compassion satisfaction levels and vice versa. Therefore, initiatives to increase nurses' compassion satisfaction levels may serve as a buffer against compassion fatigue [26, 27].

Marital status predicts burnout and compassion satisfaction among nurses in Korea [5, 26]. The variations in nurses' burnout, secondary traumatic stress, and compassion satisfaction may be explained by the effects of cultural differences and perceptions on spouses’ social support [28]. Nurses’ social support derived from their spouse or children could mitigate the risk of developing higher levels of burnout and increase nurses’ level of compassion satisfaction. In a study conducted by Roney & Acri (2018), female participants were optimistic about helping others; hence, sex influences compassion satisfaction and compassion fatigue levels [29].

The secondary traumatic stress experienced by nurses was predicted by the health and well-being domain. Nurses who perceive that they have better health have lower levels of secondary traumatic stress. This finding aligns with the research conducted by Stacey et al. (2016) [30]. Organizations should conduct activities that promote the mental health and well-being of nurses, such as forums or mental health weeks for nurses. Additionally, developing physical fitness initiatives such as granting nurses access to the hospital rehabilitation gymnasium before or after work, engaging the rehabilitation team to conduct group exercise workout sessions for nurses, and providing free health screening and health insurance coverage for nurses will further increase nurses’ health and well-being. Conversely, nurses who experienced greater pressure in their work domain and work‒individual interaction domain also experienced higher levels of secondary traumatic stress. High-quality patient care depends on lowering nurses' levels of compassion fatigue and increasing their levels of compassion satisfaction. Organizations should also explore the use of artificial intelligence, such as the use of robots in the nursing environment, or enhance the existing health information system to reduce nurses’ demands at work, thus reducing compassion fatigue.

Elucidating the components that contribute to compassion fatigue and compassion satisfaction might enhance the productivity and job happiness of nurses while furnishing management with substantiation to initiate remedial measures. The development or adaptation of a compassion model is the first step that organizations and the nursing administration can take to develop programmes suitable to promote compassion satisfaction and reduce compassion fatigue among nurses. Furthermore, management should take note of the health and well-being domain and the demands at work domain, which were shown to significantly predict compassion satisfaction and compassion fatigue among nurses. Staff welfare strategies such as support groups and interventions such as flexible work shifts to reduce demands at work could be explored, developed and implemented to alleviate compassion fatigue and increase compassion satisfaction among nurses.

It is important to incorporate compassionate care into the current nursing curriculum. A curriculum for providing compassionate care should be a part of clinical teaching methodologies, and it should be followed by curricular assessment to prepare future nurses for the emotional demands of the profession. For example, lecturers should encourage student nurses to develop interpersonal communication skills, which include attentive listening, empathetic language and conflict resolution. Role playing and simulation exercises provide opportunities for nurses to experience situations that require compassionate care. Additionally, nurses should regularly participate in educational programmes on compassion fatigue and compassion fulfilment. This will raise awareness among nurses and assist them in determining the major risk factors for compassion fatigue and satisfaction.

Although this study has several implications for nursing practice, it has several limitations. The cross-sectional nature of this study prevents us from making causal inferences. A mixed-method or qualitative study that is representative of all nurses in Malaysia would benefit the existing body of knowledge soon. Interventional-based studies on nurses’ personal and work environment-related factors influencing compassion satisfaction and compassion fatigue among nurses in Malaysia are needed. The use of a self-report questionnaire for data collection may also increase the risk of bias. Another drawback of this study is that the nurses may have felt uncomfortable answering the questionnaire prior to this profession, which puts them under additional pressure and stress. To reach more accurate conclusions, a larger geographical region that takes this predictor into account should be studied.

## Conclusions

The aim of this study was to understand nurses’ compassion satisfaction and compassion fatigue levels in Malaysia. Compassion fatigue is a serious concern for nurses, but it is preventable. The findings obtained provide valuable insights, as there may be detrimental effects on the healthcare industry and retention of nurses if no action is taken to combat compassion fatigue. The study revealed that the majority of nurses had decreased compassion satisfaction and increased burnout. Nurses’ work-related demands and their own perceptions of health and well-being predicted their levels of compassion satisfaction, burnout and secondary traumatic stress. Recommendations to motivate nurses and reduce demands at work by introducing measures such as flexible shift hours should be explored by healthcare organizations to increase nurses’ performance. Nursing management and nurses should take a holistic approach to improve job satisfaction and quality of care by maintaining a conducive work environment and promoting mental health awareness among nurses. By prioritizing self-care, building resilience, and fostering a supportive work environment, nurses can protect their emotional and physical well-being.

## Data Availability

Data is provided within the manuscript, figure, tables and supplementary files.

## Abbreviations

ProQOL: Professional quality of life questionnaire
COPSOQ: Copenhagen psychosocial questionnaire

## References

[1] Sacco TL, Copel LC. Compassion satisfaction: A concept analysis in nursing. Nurs Forum. 2018;53(1):76–83. 10.1111/nuf.12213. 28662300 10.1111/nuf.12213

[2] Mattioli D. Focusing on the Caregiver: Compassion Fatigue Awareness and Understanding. MEDSURG Nursing. 2018;27(5):323–7 Retrieved from http://search.ebscohost.com/login.aspx?direct=true&db=ccm&AN=132180605&site=ehost-live.

[3] Kelly L, Todd M. Compassion Fatigue and the Healthy Work Environment. AACN Advanced Critical Care. 2017;28(4):351–8. 10.4037/aacnacc2017283. 29212642 10.4037/aacnacc2017283

[4] Kelly L, Runge J, Spencer C. Predictors of Compassion Fatigue and Compassion Satisfaction in Acute Care Nurses. J Nurs Scholarsh. 2015;47(6):522–8. 10.1111/jnu.12162. 26287741 10.1111/jnu.12162

[5] Kim K, Han Y, Kwak Y, Kim JS. Professional Quality of Life and Clinical Competencies among Korean Nurses. Asian Nurs Res. 2015;9(3):200–6. 10.1016/j.anr.2015.03.002. 10.1016/j.anr.2015.03.002 26412623

[6] Pfaff KA, Freeman-Gibb L, Patrick LJ, DiBiase R, Moretti O. Reducing the “cost of caring” in cancer care: Evaluation of a pilot interprofessional compassion fatigue resiliency program. J Interprof Care. 2017;31(4):512–9. 10.1080/13561820.2017.1309364. 28471255 10.1080/13561820.2017.1309364

[7] Hunsaker S, Chen HC, Maughan D, Heaston S. Factors That Influence the Development of Compassion Fatigue, Burnout, and Compassion Satisfaction in Emergency Department Nurses. J Nurs Scholarsh. 2015;47(2):186–94. 10.1111/jnu.12122. 25644276 10.1111/jnu.12122

[8] Al-Majid S, Carlson N, Kiyohara M, Faith M, Rakovski C. Assessing the Degree of Compassion Satisfaction and Compassion Fatigue Among Critical Care, Oncology, and Charge Nurses. J Nurs Adm. 2018;48(6):310–5. 10.1097/NNA.0000000000000620. 29794595 10.1097/NNA.0000000000000620

[9] Cetrano G, Tedeschi F, Rabbi L, Gosetti G, Lora A, Lamonaca D, Amaddeo F. How are compassion fatigue, burnout, and compassion satisfaction affected by quality of working life? Findings from a survey of mental health staff in Italy. BMC Health Serv Res. 2017;17(1):755. 10.1186/s12913-017-2726-x. 29162095 10.1186/s12913-017-2726-x PMC5696765

[10] Salimi S, Pakpour V, Rahmani A, Feizollahzadeh H, Wilson M. Compassion Satisfaction, Burnout, and Secondary Traumatic Stress Among Critical Care Nurses in Iran. J Transcult Nurs. 2020;31(1):59–66. 10.1177/1043659619838876. 30957715 10.1177/1043659619838876

[11] Hamilton S. Compassion fatigue: the cost of caring. Emergency Nurse New Zealand. 2018;4:6–7. Retrieved from: http://search.ebscohost.com/login.aspx?direct=true&db=ccm&AN=132862633&site=ehost-live.

[12] Lanier J, Brunt B. Running on Empty: Compassion Fatigue in Nurses and Non-Professional Caregivers. ISNA Bulletin. 2019;45(3):10–5 Retrieved from: http://search.ebscohost.com/login.aspx?direct=true&db=ccm&AN=136258825&site=ehost-live.

[13] Pillay S. Will Malaysia face a shortage of nurses by 2020? New Straits Times. 2017, January 1. Retrieved from https://www.nst.com.my/news/2017/01/201014/will-malaysia-face-shortage-nurses-2020.

[14] Aisyahton S, Zamzaliza AM, Siti Khuzaimah AS, Wan Hartini WZ. Factors contributed to job satisfaction among nurses working at tertiary hospitals in the Klang Valley: An adaptation of the Herzberg’s Theory. J Sustain Sci Manag. 2023;18(6):135-148.eISSN: 2672-7226.

[15] Stamm BH. The concise ProQOL manual. 2nd edition. Pocatello; 2010. Retrieved from ProQOL.org.

[16] Von EE, Altman DG, Egger M, Pocock SJ, Gotzsche PC, Vandenbroucke JP. Strengthening the Reporting of Observational Studies in Epidemiology (STROBE) statement: Guidelines for reporting observational studies. BMJ. 2007;335:806–8. 17947786 10.1136/bmj.39335.541782.AD PMC2034723

[17] Wang J, Okoli CTC, He H, Feng F, Li J, Zhuang L, Lin M. Factors associated with compassion satisfaction, burnout, and secondary traumatic stress among Chinese nurses in tertiary hospitals: A cross-sectional study. Int J Nurs Stud. 2020;102: 103472. 10.1016/j.ijnurstu.2019.103472. 31810017 10.1016/j.ijnurstu.2019.103472

[18] Useche SA, Montoro L, Alonso F, Pastor JC. Psychosocial Work Factors, Job Stress and Strain at the Wheel: Validation of the Copenhagen Psychosocial Questionnaire (COPSOQ) in Professional Drivers. Frontier in Psychology. 2019;10:1531. 10.3389/fpsyg.2019.01531. 10.3389/fpsyg.2019.01531 PMC6614297 31312166

[19] Stamm BH. ProQOL Tool for Self-Assessment of ProQOL. Professional Quality of Life: Compassion Satisfaction and Fatigue Version 5(ProQOL). 2009; 264–66. Available from: https://socialwork.buffalo.edu/content/dam/socialwork/home/self-care-kit/compassion-satisfaction-and-fatigue-stamm-2009.pdf.

[20] Axisa C, Nash L, Kelly P, Willcock S. Burnout and distress in Australian physician trainees: Evaluation of a wellbeing workshop. Australas Psychiatry. 2019;27(3):255–61. 10.1177/1039856219833793. 30854868 10.1177/1039856219833793

[21] Tseng HM, Shih WM, Shen YC, Ho LH, Wu CF. Work Stress, Resilience, and Professional Quality of Life Among Nurses Caring for Mass Burn Casualty Patients After Formosa Color Dust Explosion. J Burn Care Res. 2018;39(5):798–804. 10.1093/jbcr/irx053. 29931121 10.1093/jbcr/irx053

[22] Zhang YY, Han WL, Qin W, Yin HX, Zhang CF, Kong C, Wang YL. Extent of compassion satisfaction, compassion fatigue and burnout in nursing: A meta-analysis. J Nurs Manag. 2018;26(7):810–9. 10.1111/jonm.12589. 30129106 10.1111/jonm.12589

[23] Burr H, Berthelsen H, Moncada S, Nübling M, Dupret E, Demiral Y, et al. The third version of the Copenhagen Psychosocial Questionnaire. Paper presented at the 13th European Academy of Occupational Health Psychology Conference. Lisbon: EAOHP; 2018.

[24] Stamm BH. ProQOL Tool for Self-Assessment of ProQOL. Professional Quality of Life: Compassion Satisfaction and Fatigue Version 5 (ProQOL). 2009; 264–66. Available from: https://socialwork.buffalo.edu/content/dam/socialwork/home/self-care-kit/compassion-satisfaction-and-fatigue-stamm-2009.pdf.

[25] Khamisa N, Peltzer K, Ilic D, Oldenburg B. Work related stress, burnout, job satisfaction and general health of nurses: a follow-up study. Int J Nurs Pract. 2016;22:538–45. 10.1111/ijn.12455. 27241867 10.1111/ijn.12455

[26] Lee H, Baek W, Lim A, Lee D, Pang Y, Kim O. Secondary traumatic stress and compassion satisfaction mediate the association between stress and burnout among Korean hospital nurses: a cross-sectional study. BMC Nurs. 2021;20(1):1–10. 34193135 10.1186/s12912-021-00636-w PMC8243298

[27] Wijdenes KL, Badger TA, Sheppard KG. Assessing Compassion Fatigue Risk among Nurses in a Large Urban Trauma Center. J Nurs Adm. 2019;49(1):19–23. 10.1097/NNA.0000000000000702. 30499866 10.1097/NNA.0000000000000702

[28] Dilmaghani RB, Armoon B, Moghaddam LF. Work-family conflict and the professional quality of life and their sociodemographic characteristics among nurses: a cross-sectional study in Tehran. Iran BMC Nursing. 2022;21(1):1–9. 10.1186/s12912-022-01069-9. 10.1186/s12912-022-01069-9 PMC9624043 36316741

[29] Roney LN, Acri MC. The Cost of Caring: An Exploration of Compassion Fatigue, Compassion Satisfaction, and Job Satisfaction in Pediatric Nurses. J Pediatr Nurs. 2018;40:74–80. 10.1016/j.pedn.2018.01.016. 29402658 10.1016/j.pedn.2018.01.016

[30] Stacey W, Singh-Carlson S, Odell A, Reynolds G, Yuhua S. Compassion Fatigue, Burnout, and Compassion Satisfaction Among Oncology Nurses in the United States and Canada. Oncol Nurs Forum. 2016;43(4):E161–9. 10.1188/16.ONF.E161-E169. 27314199 10.1188/16.ONF.E161-E169

